# Ontogenetic variability of the Siberian silk moth, *Dendrolimus sibiricus*

**DOI:** 10.3389/finsc.2026.1768865

**Published:** 2026-05-21

**Authors:** Y. B. Akhanaev, M. E. Yakimova, S. V. Pavlushin, D. D. Kharlamova, A. V. Kolosov, A. A. Ageev, N. I. Ershov, V. V. Martemyanov

**Affiliations:** 1 Sirius University, Sochi, Russia; 2 Institute of Systematics and Ecology of Animals, Novosibirsk, Russia; 3 State Research Center of Virology and Biotechnology “VECTOR”, Novosibirsk, Russia; 4 All-Russian Research Institute of Forestry and Forestry Mechanization (VNIILM), “Forest Pyrology Center”, Krasnoyarsk, Russia; 5 Institute of Cytology and Genetics, Novosibirsk, Russia

**Keywords:** diapause-like, forest pest, Lepidoptera, life cycle, transcriptome

## Abstract

**Introduction:**

The Siberian silk moth *Dendrolimus sibiricus* is a major boreal defoliator whose outbreak dynamics vary with the proportion of larvae following 1-year versus 2-year life cycles, yet the physiological basis of this ontogenetic variability remains unclear.

**Methods:**

We generated tissue-specific RNA sequencing datasets from male and female larvae expressing 1-year or 2-year life cycles, dissected into head, gut, and fat body tissue sets (49 libraries), and quantified life-cycle effects using principal component analysis and differential expression with functional enrichment.

**Results:**

Across tissue sets, transcriptomic profiles were structured primarily by life cycle and secondarily by sex, with the clearest sex-dependent signal confined to gut tissues. The 2-year life-cycle trajectory was characterized by broad down-regulation of genes associated with growth and anabolic metabolism, and up-regulation of stress tolerance, catabolic remodeling, proteasome-related functions, and detoxification modules.

**Discussion:**

In *D. sibiricus* larvae expressing the 2-year life cycle, transcriptomic patterns indicate that the prolonged larval development underlying this trajectory is associated with a diapause-like state, based on similarities to diapause-associated molecular patterns described in other insects. Laboratory manipulations of larval density, food deprivation, and host plant species did not induce a switch to the 2-year life cycle, suggesting that life-cycle determination depends on more complex cue integration and/or environmental history. Together, these results identify candidate molecular signatures of life-cycle state in *D. sibiricus* and provide a foundation for future studies of life-cycle regulation in this species.

## 1 Introduction

The Siberian silk moth, *Dendrolimus sibiricus* Tsch. 1908 (Lepidoptera: Lasiocampidae), is one of the most important pests of boreal forests in the Palearctic zone, causing significant damage to various coniferous trees, including *Abies*, *Picea*, *Larix*, and *Pinus* species (1, 2). Its native range covers the eastern regions of European Russia, all of Asian Russia, and parts of Mongolia and northern China, where large-scale outbreaks frequently occur (3–6). The species has also been recorded in both South and North Korea (7). Phylogenetic analyzes based on mtDNA and nuclear ITS2 markers place *D. sibiricus* in a species complex with *D. pini* and *D. superans*, indicating a relatively recent divergence from *D. superans*, with evidence of mitochondrial introgression into western *D. pini* populations (8). *Dendrolimus sibiricus* outbreaks lead to substantial tree mortality (9, 10), inducing significant alterations in forest structure. An increase in wildfire frequency and area burned has been linked to defoliation by this pest (11). Moreover, *D. sibiricus* can develop on non-native conifer hosts (12), suggesting potential invasion risks in new regions (13, 14). Climate-driven northward range shifts have already been observed (15), and the moth’s ability to utilize novel hosts may accelerate its spread into previously unaffected forests. This possibility underscores the need for monitoring and control measures to mitigate ecological and economic impacts in both native and non-native regions.

**Figure 1:**
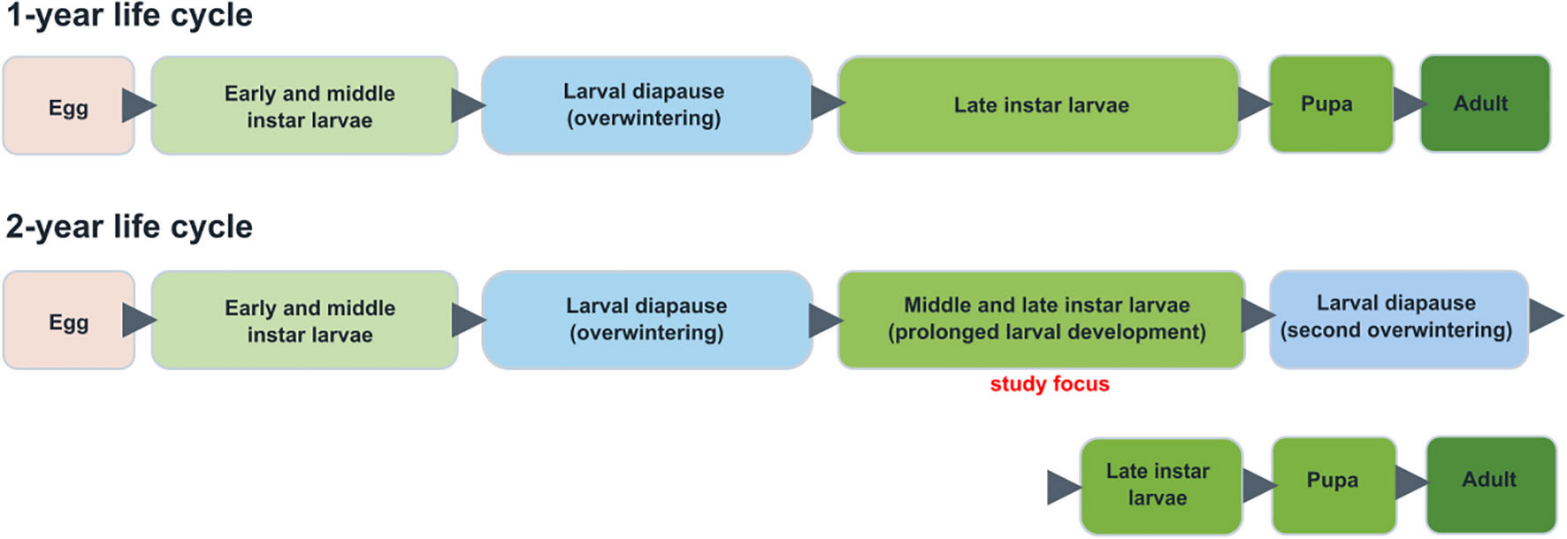
Schematic representation of naturally occurring 1-year and 2-year life cycles in *D. sibiricus*.

Diapause is a programmed developmental arrest that is initiated before the onset of adverse conditions and plays a central role in seasonal adaptation in insects (16). In species with larval diapause, developmental delay may include both classical diapause and prolonged larval development associated with markedly reduced growth and metamorphic progression (17). In *D. sibiricus*, larval overwintering is a regular component of development. Typically, *D. sibiricus* completes development from egg to adult within two calendar years (18), commonly referred to as a 1-year life cycle because larvae overwinter once (**Figure 1**). Eggs are laid on host tree needles, and first instar larvae feed for about 3–4 weeks. By September, second or early third instar larvae descend to the forest litter and overwinter. Larvae resume feeding the following spring, continue developing, and pupate (**Figure 1**). Under some environmental conditions, however, a subset of larvae experiences an extended larval stage, prolonging development through the summer months. These larvae descend again in autumn for a second overwintering, thus exhibiting a 2-year life cycle (19). In this study, we focused specifically on these 2-year life cycle larvae. The most severe tree defoliation occurs the following spring, when larvae from both 1- and 2-year cycles simultaneously resume feeding. The synchronized pupation and adult emergence of both cycles results in a marked increase in insect numbers.

Most previous studies have focused on the general biology, population dynamics, and ecological consequences of *D. sibiricus* outbreaks (5, 15, 20, 21). Despite this, the physiological and molecular mechanisms underlying *D. sibiricus* life-cycle variability remain poorly understood. Investigating the mechanisms behind the transition between 1- and 2-year life cycles is important for understanding the biology of life-cycle variation in *D. sibiricus* and may also yield broader insights into insect diapause and developmental plasticity (22). Genomic resources in boreal Lepidoptera illustrate the value of molecular approaches for studying overwintering-related traits and outbreak biology (23, 24). These studies underscore how genomic and transcriptomic approaches can illuminate the mechanisms underlying overwintering capacity and outbreak dynamics in forest Lepidoptera.

Recently, the whole genome of *D. sibiricus* was sequenced (NCBI accession GCA_046627935.1), providing genomic resources that support transcriptome-based analyzes of life-cycle-associated molecular variation. This offers an opportunity to explore the genetic regulation of life-cycle variability in *D. sibiricus*. In this study, we evaluated transcriptomic data to identify differentially expressed genes (DEGs) between larvae exhibiting 1- and 2-year life cycles in *D. sibiricus*. To separate sources of expression variation, larvae from both life-cycle variants were dissected into three tissue sets: (1) head capsule plus first two body segments; (2) gut with gonads and Malpighian tubules; and (3) fat body with cuticle and remaining organs, for RNA sequencing. Our goal was to elucidate the transcriptional mechanisms underlying the two life-cycle patterns. In addition, we conducted laboratory experiments to test whether certain factors could trigger the 2-year life cycle. Specifically, we examined the effects of population density, food deprivation, and different host plant species on the incidence of prolonged larval development (2-year life cycle). By rearing larvae under controlled conditions while varying these factors, we aimed to identify stimuli that induce a developmental delay characteristic of the 2-year life cycle. Consequently, this mechanistic understanding may ultimately contribute to improved understanding of life-cycle variation relevant to monitoring and management. It also allows more informed evaluation of how different pest population management scenarios may influence the timing and magnitude of defoliation.

## 2 Materials and methods

### 2.1 Insects and sample preparation

Larvae of *D. sibiricus* were collected in the Irkutsk region (53.23°N, 101.1°E) of Russia. The primary host plant for these larvae was Siberian pine (*Pinus sibirica*). Larvae were initially placed in ventilated plastic containers (~50 larvae in a 20 L container) and fed fresh host plant needles inserted into water-filled tubes sealed with parafilm to prevent water loss. Subsequently, larvae were reared on Siberian larch (*Larix sibirica*) branches under controlled laboratory conditions (23 °C constant temperature, 16:8 photoperiod) until pupation. Emerging adults were transferred to 1 L plastic containers (at a ratio of 1♀:2♂) containing paper substrate and fresh larch branches for mating and oviposition. Moist cotton wool was placed at the bottom of the container and rehydrated regularly. Eggs were collected into Petri dishes on moistened cotton wool (~100 eggs per dish) and incubated at 25 °C until hatching (approximately 14–19 days). Neonate larvae were then transferred to 20 L containers and reared on Siberian larch under the same laboratory conditions.

During laboratory rearing, marked differences in larval body mass and instar were observed among the *D. sibiricus* larvae. Larvae used for transcriptomic analysis were sampled after this divergence had become evident. Fourth- and fifth-instar larvae whose body mass was approximately twice that of conspecifics from the same containers were classified as expressing a 1-year life cycle. Larvae in the third instar on the same date were classified as having a 2-year life cycle. Instars were determined by head capsule width following Rozhkov (2). Larval sex was determined morphologically during dissection by examining gonad shape (2).

For transcriptomic analyzes, larvae from both life-cycle variants (1-year and 2-year) were dissected into three tissue sets representing major endocrine, digestive, and storage/metabolic compartments. The head set comprised the head capsule and first two body segments, including the brain and endocrine glands that regulate juvenile hormone (JH) and ecdysteroid titers during diapause and metamorphosis (25). The gut set comprised the gut, gonads, and Malpighian tubules and was chosen to capture changes in the digestive and reproductive organs, given that previous work on *D. sibiricus* showed pronounced differences in feeding and assimilation between continuously developing and development-prolonged larvae on different conifer hosts (19). The fat body set comprised the fat body, cuticle, and remaining internal organs, capturing systemic metabolic and storage responses; the fat body is the principal organ of energy storage and mobilization during diapause (22, 25). Each dissected tissue set from an individual larva was placed in a 1.5 mL microcentrifuge tube, immediately frozen in liquid nitrogen, and stored at –80 °C until RNA extraction. RNA was extracted from individual larvae and sequenced separately. The head dataset comprised 11 larvae (1-year: 3 females and 2 males; 2-year: 3 females and 3 males), the fat body dataset comprised 18 larvae (1-year: 5 females and 4 males; 2-year: 4 females and 5 males), and the gut dataset comprised 20 larvae (1-year: 4 females and 5 males; 2-year: 5 females and 6 males).

### 2.2 RNA extraction and sequencing

Frozen samples were homogenized by grinding. Total RNA was extracted from each sample using PureZOL™ RNA Isolation Reagent (Bio-Rad #732-6890) according to the manufacturer’s instructions. To eliminate DNA contamination, RNA samples were treated with DNase I and then purified using Agencourt RNA Clean XP magnetic beads, with the RNA finally eluted in nuclease-free water.

RNA concentration and purity were assessed via a NanoDrop 2000 spectrophotometer. Samples with A260/A280 and A260/A230 ratios exceeding 1.8 were deemed suitable for sequencing. Non-strand-specific poly(A) mRNA libraries were prepared and sequenced on an Illumina NovaSeq platform (performed by Novogene).

### 2.3 Differential gene expression analysis

Raw RNA-seq reads were evaluated with FastQC v0.11.9 https://www.bioinformatics.babraham.ac.uk/projects/fastqc/ and Picard v2.27.1 https://broadinstitute.github.io/picard/ for quality control. Reads were mapped to the *D. sibiricus* reference genome (NCBI accession GCA_046627935.1) using STAR v2.6.1e https://github.com/alexdobin/ STAR with a two-pass alignment strategy (the second pass mapping incorporating splice junctions detected in the first pass). The STAR genome index was built without providing a gene annotation. Gene-level read counts were obtained using featureCounts (Subread v2.0.6) (26) in R v4.5.1 (27) with parameters --primary -s 0 -p --countReadPairs.

Differential expression analysis was carried out in DESeq2 v1.48.2 (28). Genes were included in the analysis only if they had ≥ 10 read counts in at least 2 samples. We fit generalized linear models (GLMs) with a negative binomial error distribution, including life cycle (1-year vs. 2-year), sex, and their interaction (life_cycle × sex) as fixed effects. The interaction term was assessed with a likelihood ratio test (LRT); if the interaction was not significant (adjusted p > 0.1), it was dropped and an additive model was used (~ life_cycle + sex). Genes were deemed significantly differentially expressed at a false discovery rate (FDR) with Benjamini–Hochberg adjusted p < 0.1 and with absolute value of |log2 fold change| > 1. We performed principal component analysis (PCA) on variance-stabilizing transformed (VST) counts obtained via the DESeq2::vst function. Overlap among significantly DEGs was visualized using Euler diagrams generated with the R package eulerr v7.0.4 (29). Volcano plots of differential expression results were generated in the R using the package ggplot2 v3.5.2 (30).

### 2.4 Functional annotation and enrichment analyzes

Functional annotations for gene sets were obtained using InterProScan v5.55-88.0 (31), querying multiple databases (InterPro v99.0, Pfam v34.0, SUPERFAMILY v1.75, TIGRFAM v15.0, PANTHER v15.0, Gene Ontology (GO) release 2023-11-15, Reactome v82). Additional functional annotation was performed with EnTAP v1.0.1 (32), querying UniProt (release 2024_02), GO (2023-11-15), and EggNOG v6.0 databases.

GO term enrichment was tested using hypergeometric tests implemented in GOstats v2.70.0 (33) on custom GeneSetCollection objects derived from InterProScan- and EnTAP-based GO annotations. Analyzes were restricted to Biological Process terms and were conducted separately for each tissue set and direction of expression change (up- and down-regulated DEGs in 2-year life cycle larvae). GO terms were considered significantly enriched at FDR < 0.05 (Benjamini–Hochberg correction). Enrichment analyzes were first performed separately for InterProScan- and EnTAP-based GO annotations; enriched GO categories from the two annotation sources were then merged by GO term identity. When a GO term was detected in both annotation sources, we retained the instance with the lower FDR (together with its corresponding odds ratio and gene count), whereas terms detected in only one pipeline were included as reported. For visualization of GO enrichment, custom dot plots were generated in R v4.5.1 (27). For each tissue set and regulation direction, the top 10 enriched GO terms were selected based on a composite score combining enrichment significance and effect size [-log10(adjusted p) × log2(odds ratio)]. Heatmaps were generated for genes associated with selected InterPro protein domains and Reactome pathways. For each tissue set, annotated genes matching the selected category were filtered against the corresponding DESeq2 results, and only genes with adjusted p < 0.05 and |log2 fold change| > 2 were retained for plotting. For gut samples, heatmaps were generated separately for females and males. Expression matrices were based on DESeq2-normalized counts, scaled by gene (row z-scores), and visualized using the pheatmap R package v1.0.13 (34).

### 2.5 Environmental factor experiments

**Figure 2:**
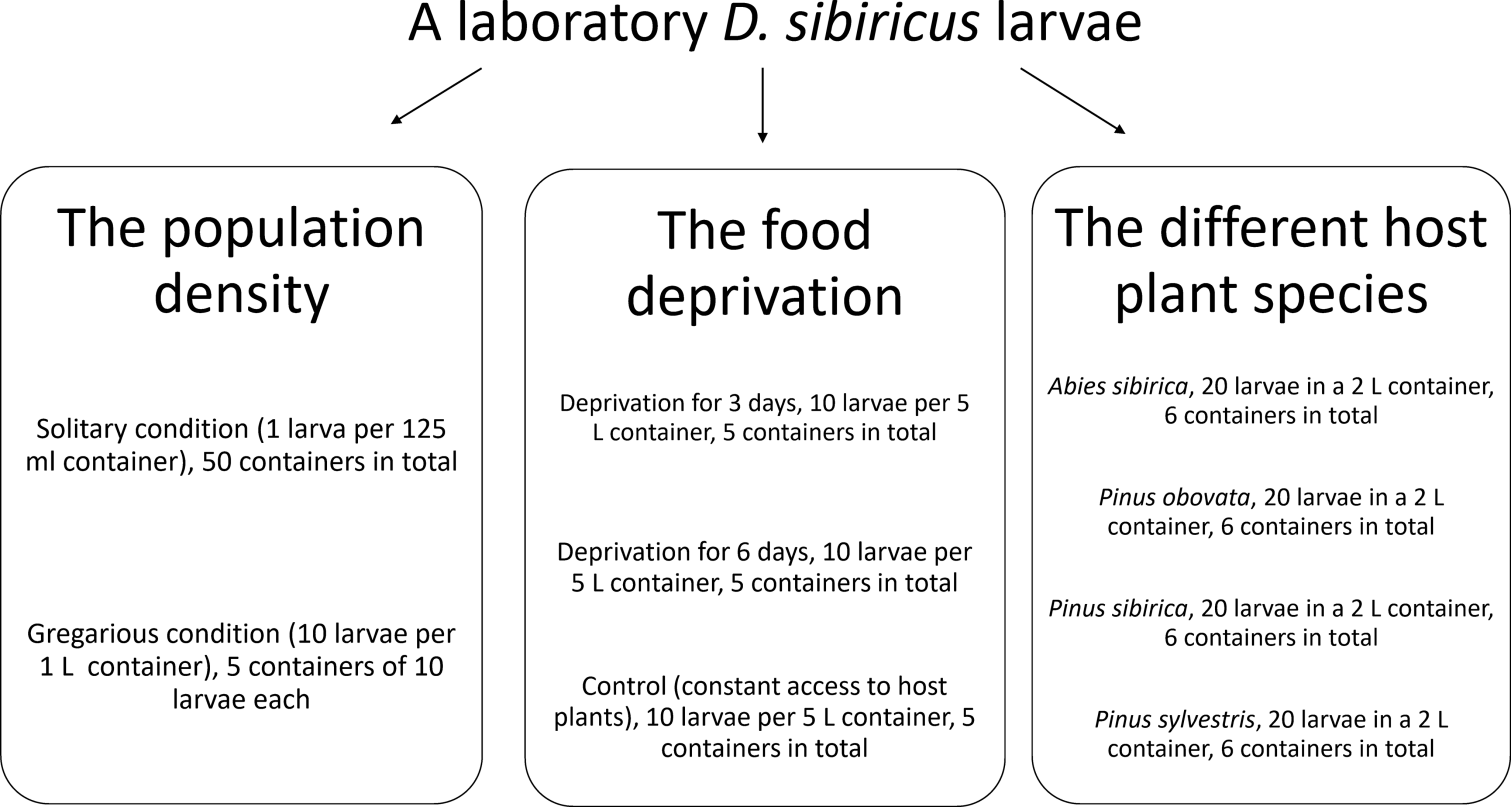
Scheme of the laboratory experiments to assess factors affecting the life cycle of *D. sibiricus*.

Additional *D. sibiricus* larvae were collected from Irkutsk (53.23°N, 101.1°E) and Altai (51.56°N, 80.26°E) regions of Russia and subsequently reared in the laboratory to examine environmental factors that determine the life cycle. We tested three factors: population density, food deprivation, and different host plant species (**Figure 2**). Insect population density effects were evaluated by a new laboratory *D. sibiricus* generation of second and third instar larvae originating from the Altai region that was reared under solitary (1/container) and gregarious (10/container) conditions. Food deprivation effects on the life cycle of *D. sibiricus* were tested on fourth instar larvae of the same laboratory *D. sibiricus* generation. To do this, the first group of insects was subjected to food deprivation by providing cut branches of Siberian larch for 24 hours every three days, while the second group was subjected to the same condition for six days (**Figure 2**). Control larvae received continuous fresh needles of Siberian larch. For host plant effects, a laboratory generation from Irkutsk was split among four hosts at hatching: Siberian fir (*Abies sibirica*), Siberian spruce (*Picea obovata*), Siberian pine (*Pinus sibirica*), and Scots pine (*Pinus sylvestris*). Since Siberian pine was the natural host plant for Irkutsk *D. sibiricus* larvae it was expected to be the most suitable. Siberian spruce and Scots pine are less preferable host plants to *D. sibiricus* larvae (12). To minimize neonate mortality on Scots pine, larvae were initially reared on Siberian pine until reaching the second instar. Larval mortality was recorded. Larval body mass was recorded at the beginning and end of the experiment and used as the primary indicator of life-cycle expression.

### 2.6 Statistical analysis

Statistical analyzes were performed in R v4.5.1 (27). Larval body mass data were non-normally distributed (Shapiro–Wilk W test, p < 0.05), so non-parametric tests were used. We compared solitary vs. gregarious larval masses with a Mann–Whitney U test. For multi-group comparisons (food deprivation, host plant species), we used Kruskal–Wallis ANOVA followed by Dunn’s *post hoc* tests. Larval mortality data were analyzed by logistic regression (GLM with binomial error and logit link).

## 3 Results

### 3.1 Transcriptome sequencing and expression patterns in *Dendrolimus sibiricus* larvae with 1- and 2-year life cycles

For both life-cycle variants of *D. sibiricus* (1-year vs. 2-year), we performed RNA-seq on male and female larvae dissected into three tissue sets (head set, fat body set, and gut set). A total of 49 cDNA libraries (BioProject accession PRJNA1451813) were sequenced and mapped to the *D. sibiricus* reference genome (GCA_046627935.1), with a mean uniquely mapped read rate of ~93%.

**Figure 3:**
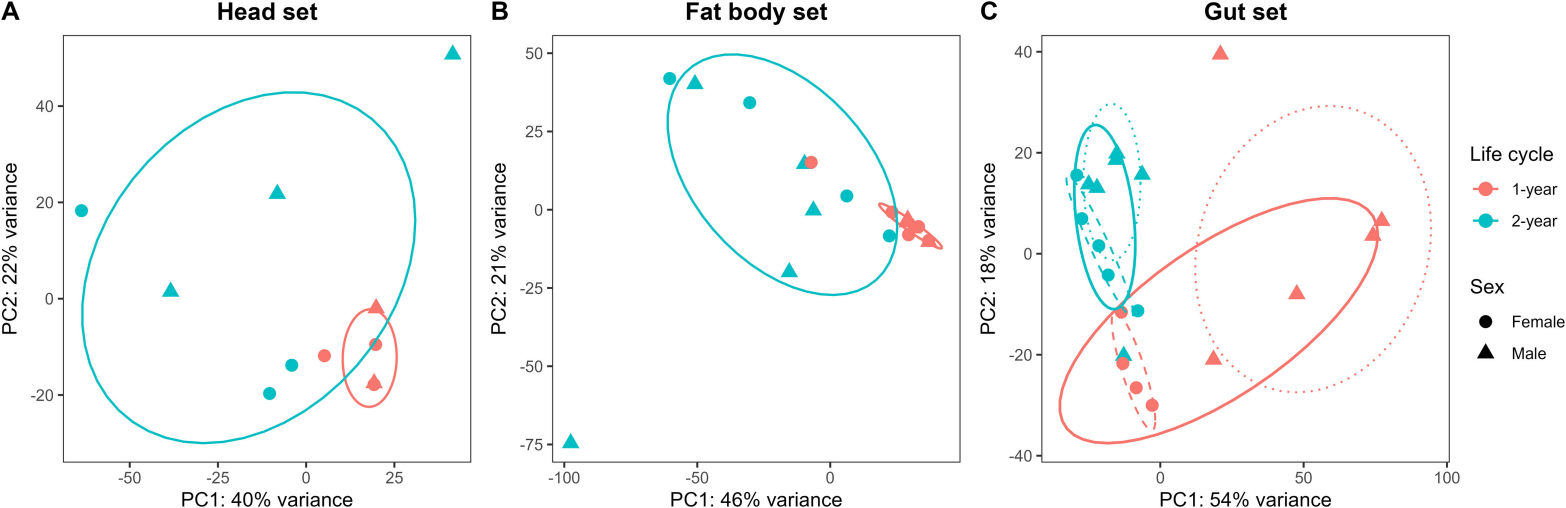
Principal component analysis of transcriptomic profiles in three tissue sets of *D. sibiricus* larvae in the head set **(A)**, the fat body set **(B)**, and the gut set **(C)**. Solid ellipses and points are colored by life cycle (1- vs. 2-year) and shaped by sex (female vs. male). Ellipses are used to illustrate group dispersion. In the panel **(C)**, additional dashed and dotted ellipses indicate females and males within the life-cycle group.

Unsupervised PCA performed for each tissue set revealed that transcriptomic variation was primarily structured by life-cycle variant (**Figures 3A–C**). Across tissue sets, PC1 accounted for 40–54% of total variance and separated samples from 1-year vs. 2-year larvae, whereas PC2 explained 18–22% of variance and captured mainly within-group variation. In the head set, the 1-year and 2-year larvae showed a clear but partially overlapping separation along PC1 (**Figure 3A**). In the fat body set, the 1-year and 2-year larvae were largely separated along PC1, indicating substantial differentiation between life-cycle groups (**Figure 3B**). In the gut set, the main separation between samples was associated with life cycle along PC1, whereas additional variation related to sex was also observed (**Figure 3C**).

In addition, the LRT revealed a substantial number of genes in the gut set with significant interaction effects: 7.26% of genes (1,046 out of 14,399). Most interaction-associated genes (n = 939) exhibited a stronger life-cycle effect in females, indicating that the interaction between life cycle and sex was predominantly driven by female-specific responses in gut tissue set. In contrast, only a small proportion of genes showed significant interaction effects in the head and fat body sets (0.15% of genes in head set, 0.31% in fat body set).

### 3.2 Life-cycle variation is associated with tissue-specific patterns of DEGs

**Figure 4:**
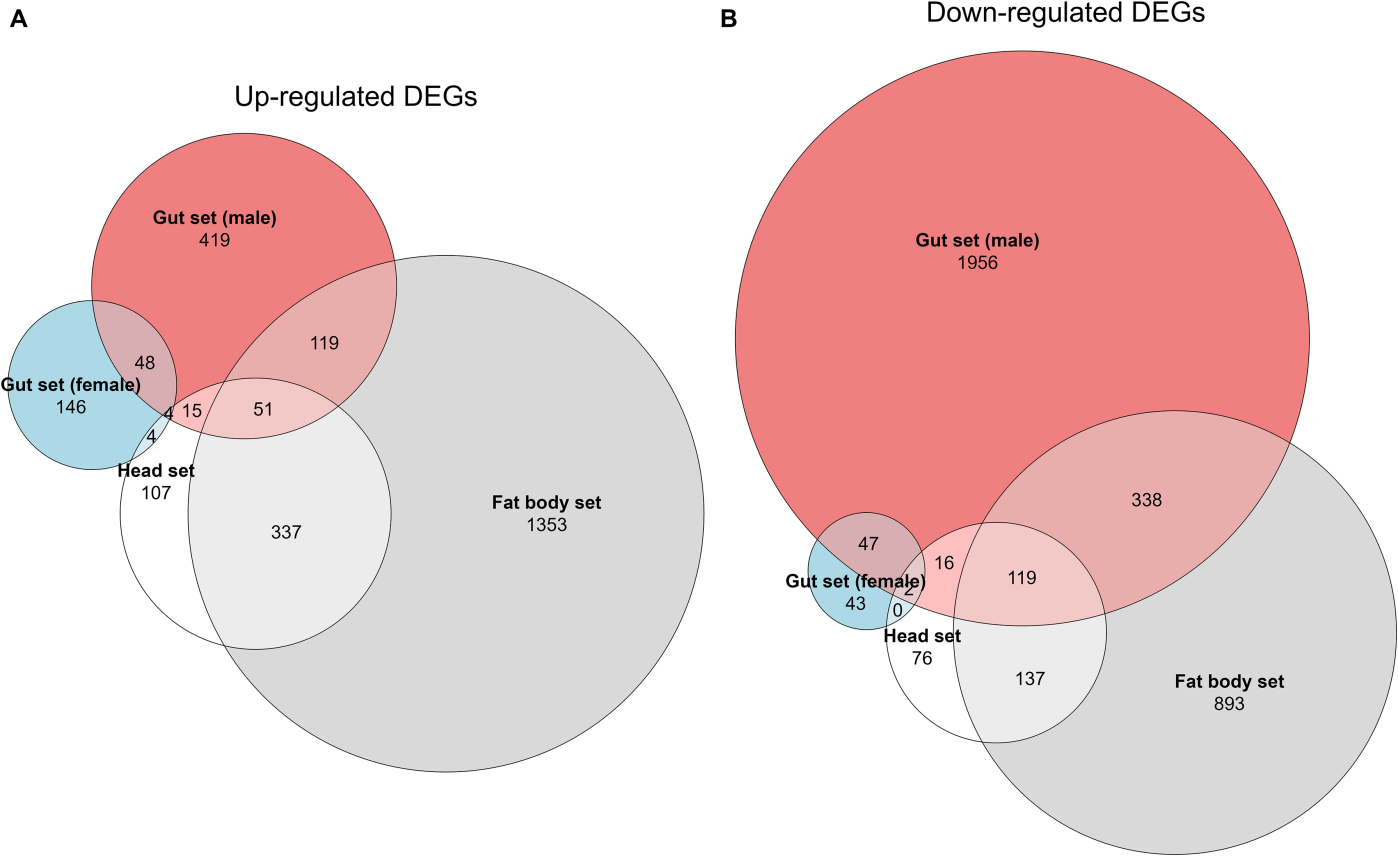
Euler diagrams showing the overlap of significantly up- **(A)** and down-regulated **(B)** DEGs identified between 1-year and 2-year life cycle variants across different tissue sets of *D. sibiricus* larvae. Circles represent the number of DEGs, with overlaps indicating shared DEGs between tissue sets.

Given the pronounced life cycle × sex interaction observed in the gut set (based on both PCA clustering (**Figure 3C**) and the LRT), we subsequently analyzed the gut data for males and females separately. Comparative transcriptomic analysis identified substantial numbers of DEGs between the 1-year and 2-year life cycles, with tissue-specific patterns of overlap among datasets (**Figures 4A, B**). A total of 6,293 DEGs were detected, with the largest number of up-regulated genes in the fat body set (**Figure 4A**) and the most down-regulated genes in the male gut set (**Figure 4B**). The overlap of DEGs between tissue sets was relatively low, indicating that gene-expression differences associated with life cycle are largely tissue-specific (**Figures 4A, B**).

**Figure 5:**
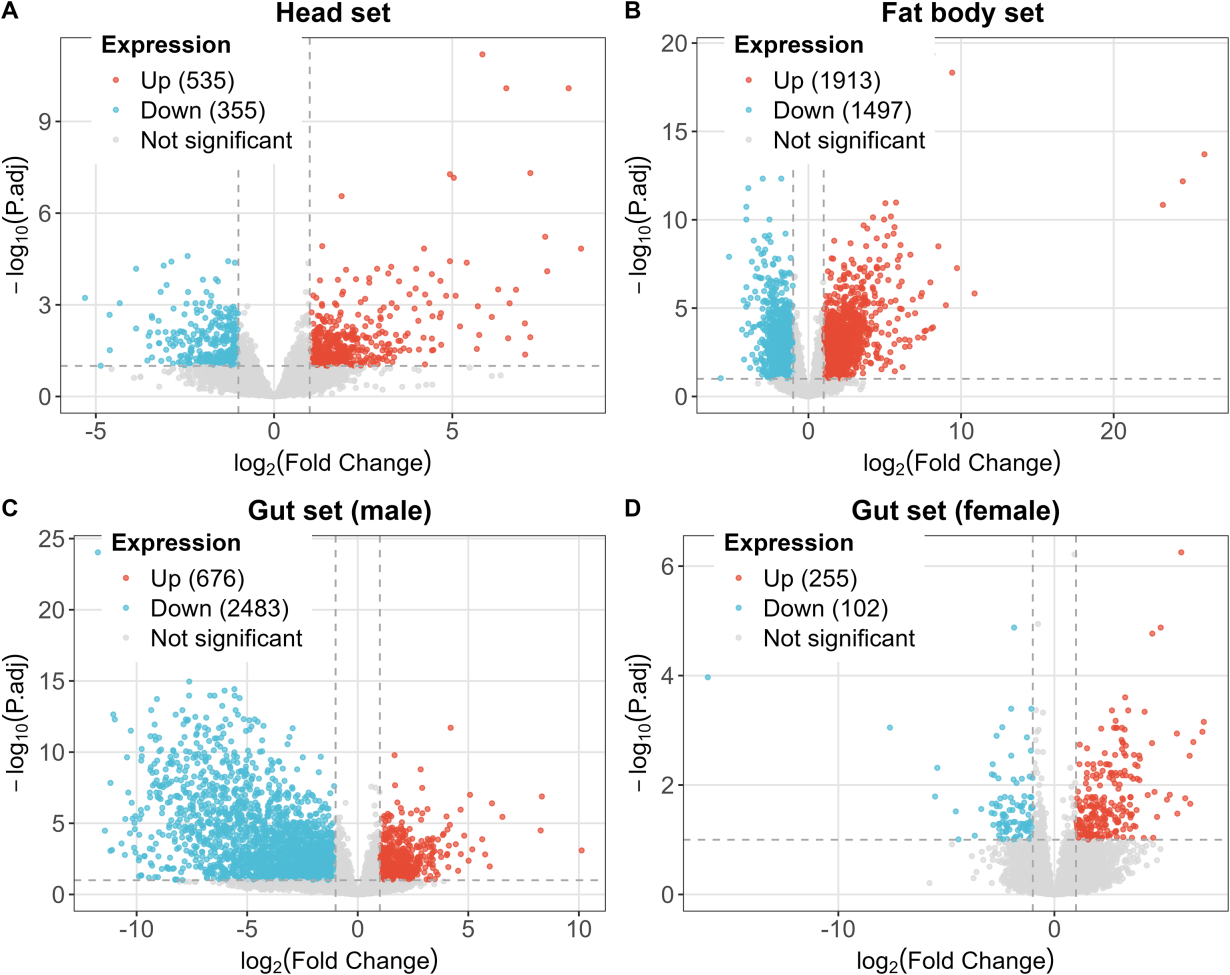
Volcano plots of DEGs between 1-year and 2-year life cycles of *D. sibiricus* larvae in head **(A)**, fat body **(B)**, and sex-separated gut sets **(C, D)**. Dashed vertical lines indicate the log2 fold-change thresholds (± 1), and the dashed horizontal line indicates the adjusted p-value threshold (0.1) used to define DEGs.

Volcano plot analysis highlighted distinct differential expression patterns among tissue sets and sexes (**Figures 5A–D**). In the head set, 890 DEGs were identified, with 535 genes significantly up-regulated and 355 down-regulated in 2-year vs. 1-year larvae (**Figure 5A**). In the fat body set, 3,410 DEGs were identified, comprising 1,913 up-regulated and 1,497 down-regulated genes in 2-year larvae (**Figure 5B**). The male gut set exhibited 3,159 DEGs (676 up-regulated, 2,483 down-regulated; **Figure 5C**). The female gut set had fewer DEGs (357 total; 255 up-regulated, 102 down-regulated; **Figure 5D**).

### 3.3 Functional significance of DEGs between life-cycle variants

**Figure 6:**
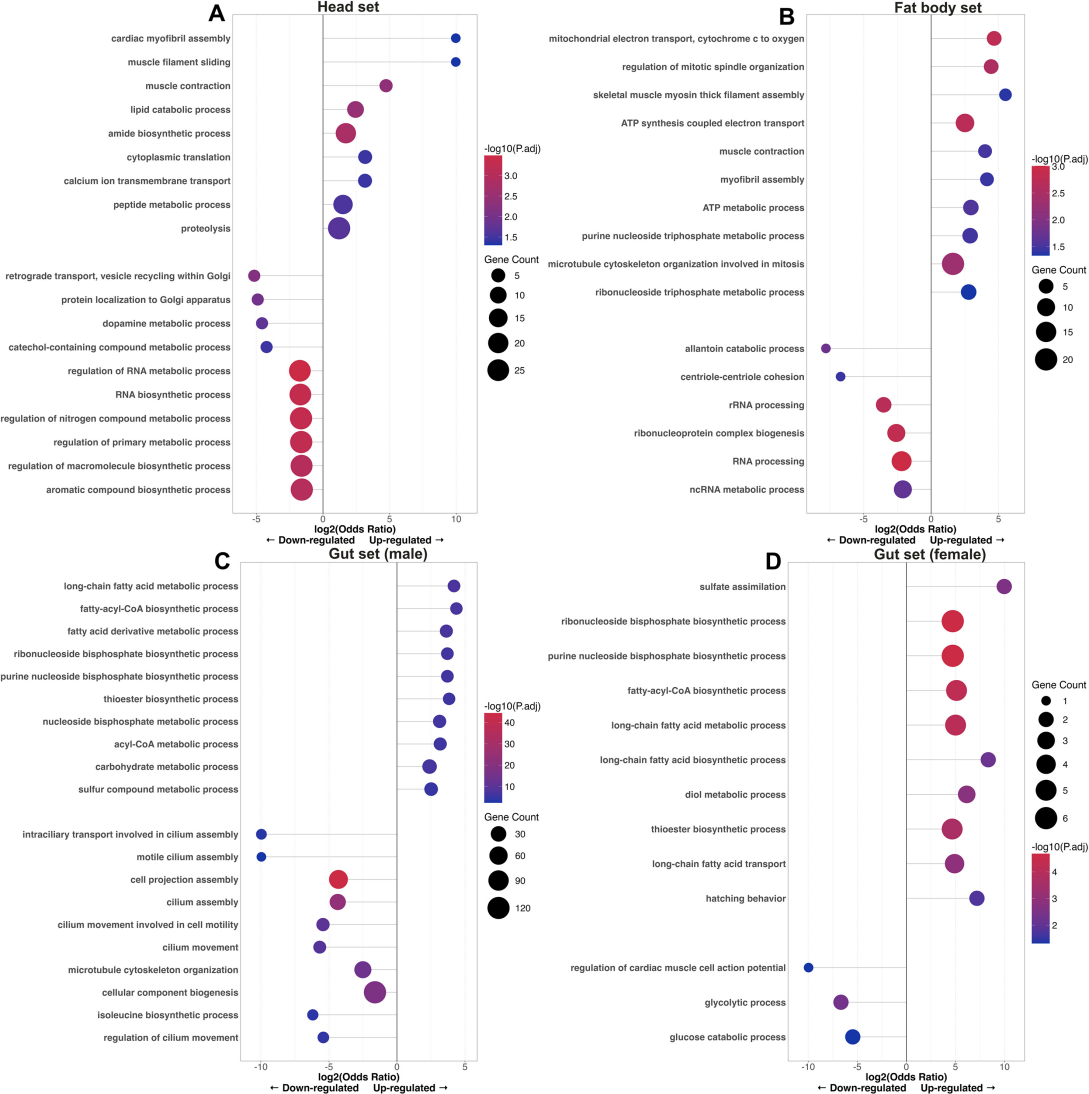
Dot plots showing enriched GO biological processes among up- and down-regulated genes in 2- vs. 1-year life cycle larvae of *D. sibiricus* across head **(A)**, fat body **(B)** and sex-separated gut sets **(C, D)**.

To explore the biological processes associated with differential gene expression between 1-year and 2-year life cycles larvae, we performed GO enrichment analysis based on InterProScan- and EnTAP-derived annotations and summarized GO categories for each tissue set (**Figures 6A–D**). The head set showed enrichment in muscle-related processes and cytoplasmic translation among genes up-regulated in 2-year life cycle larvae. Genes down-regulated in head set were primarily associated with vesicle transport, RNA biosynthesis, and dopamine metabolism (**Figure 6A**). In fat body set, genes up-regulated in 2-year life cycle larvae were linked to mitochondrial electron transport, ATP synthesis, and muscle contraction, whereas genes down-regulated were involved in ribonucleoprotein complex biogenesis, RNA processing, and rRNA processing (**Figure 6B**). In the male gut set, down-regulated genes were enriched for cilium-related terms, whereas up-regulated genes were enriched for metabolic processes (**Figure 6C**). Finally, in female gut set, genes up-regulated were enriched in biosynthetic processes, including fatty acid and nucleotide metabolism (**Figure 6D**). Overall, these GO enrichment results indicate that distinct tissue-specific biological processes underlie life-cycle variability in *D. sibiricus*, highlighting differential metabolic and structural adjustments associated with the transition from 1-year to 2-year life cycle.

### 3.4 Molecular pathway differences underlying life-cycle variability in *Dendrolimus sibiricus*

**Figure 7:**
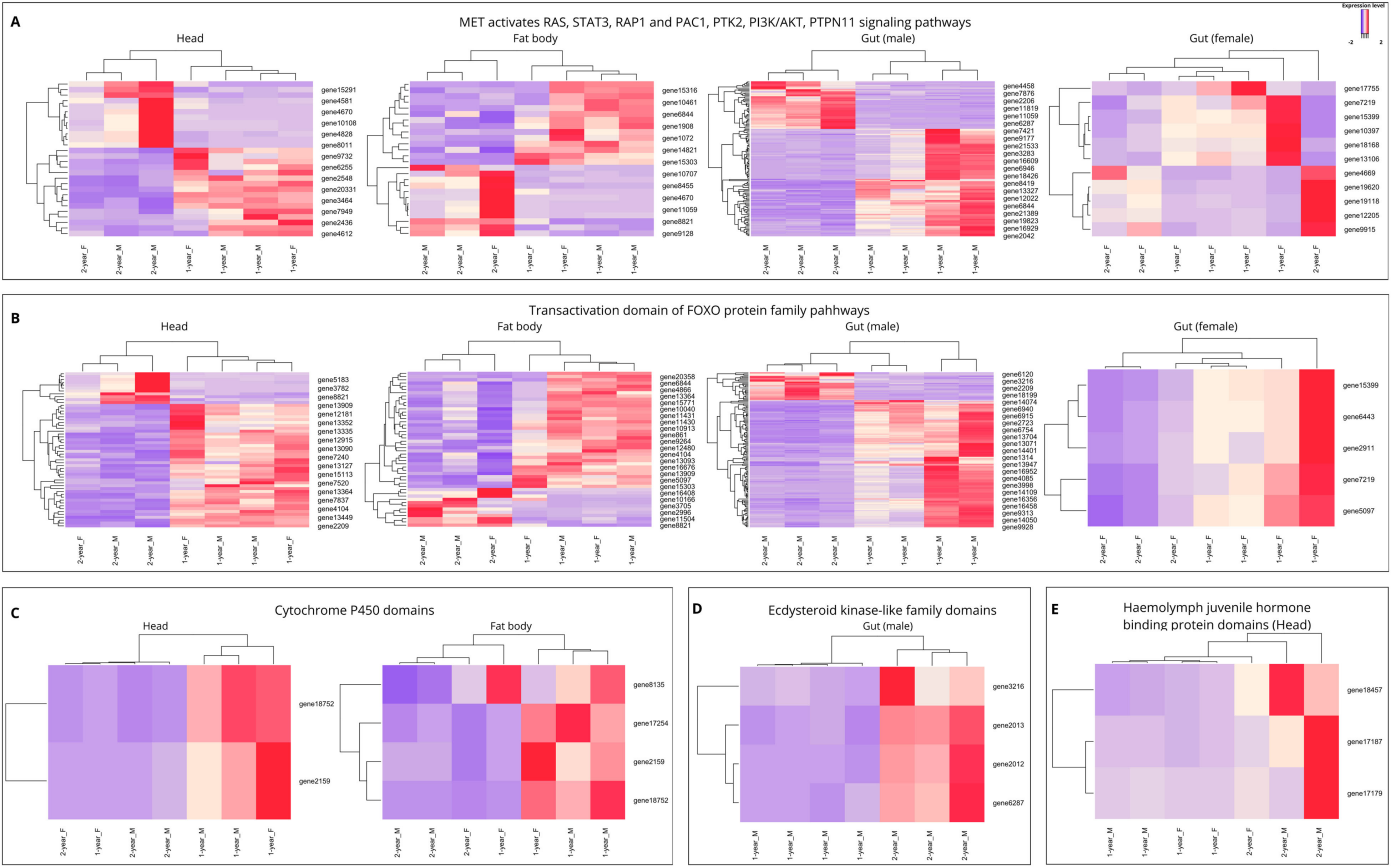
Expression heatmaps of life-cycle DEGs in key signaling and hormonal pathways across tissue sets. Heatmaps show normalized expression of strongly regulated life-cycle DEGs (|log_2_FC| > 2, padj < 0.05) belonging to selected Reactome pathways and protein domains across head, fat body and gut tissue sets. **(A)** MET activates RAS/STAT/PI3K signaling pathway. **(B)** Transactivation domain of FOXO protein family pathways. **(C)** Cytochrome P450 domains. **(D)** Ecdysteroid kinase-like family domains (gut, male). **(E)** Hemolymph juvenile hormone binding protein (JHBP) domains (head). Columns denote *D. sibiricus* life cycle larvae (1- vs. 2-year), rows represent individual DEGs within each pathway/domain.

We next examined expression patterns of DEGs assigned to selected Reactome pathways and protein domain families across head, fat body and sex-separated gut tissue sets of *D. sibiricus* larvae (**Figures 7A–E**). The Methoprene-tolerant (Met) signaling pathway was prominently represented among DEGs, highlighting critical downstream targets such as PI3K/AKT, STAT3, RAP1/RAC1, PTK2, and PTPN11 (**Figure 7A**). Forkhead box protein O1 (FOXO) signaling showed both up- and down-regulated DEGs. Although a bidirectional pattern was observed, most of the down-regulated DEGs in 2-year larvae were found across all tissue sets (**Figure 7B**). Several cytochrome P450 domain-containing genes were down-regulated in 2-year life cycle larvae, although the functional significance of this pattern remains uncertain (**Figure 7C**). Genes annotated with ecdysteroid kinase-like domains were also up-regulated in 2-year life cycle larvae (**Figure 7D**), which may be consistent with altered ecdysteroid turnover during prolonged development. Domains associated with hemolymph juvenile hormone-binding proteins (JHBP) in head set were differentially expressed between life-cycle variants. Higher expression of JHBP-related transcripts in 2-year life cycle larvae implies altered JH signaling, which may contribute to suppression of metamorphic transitions and prolonged larval development (**Figure 7E**).

### 3.5 Contribution of environmental factors to switching of the life-cycle program

**Figure 8:**
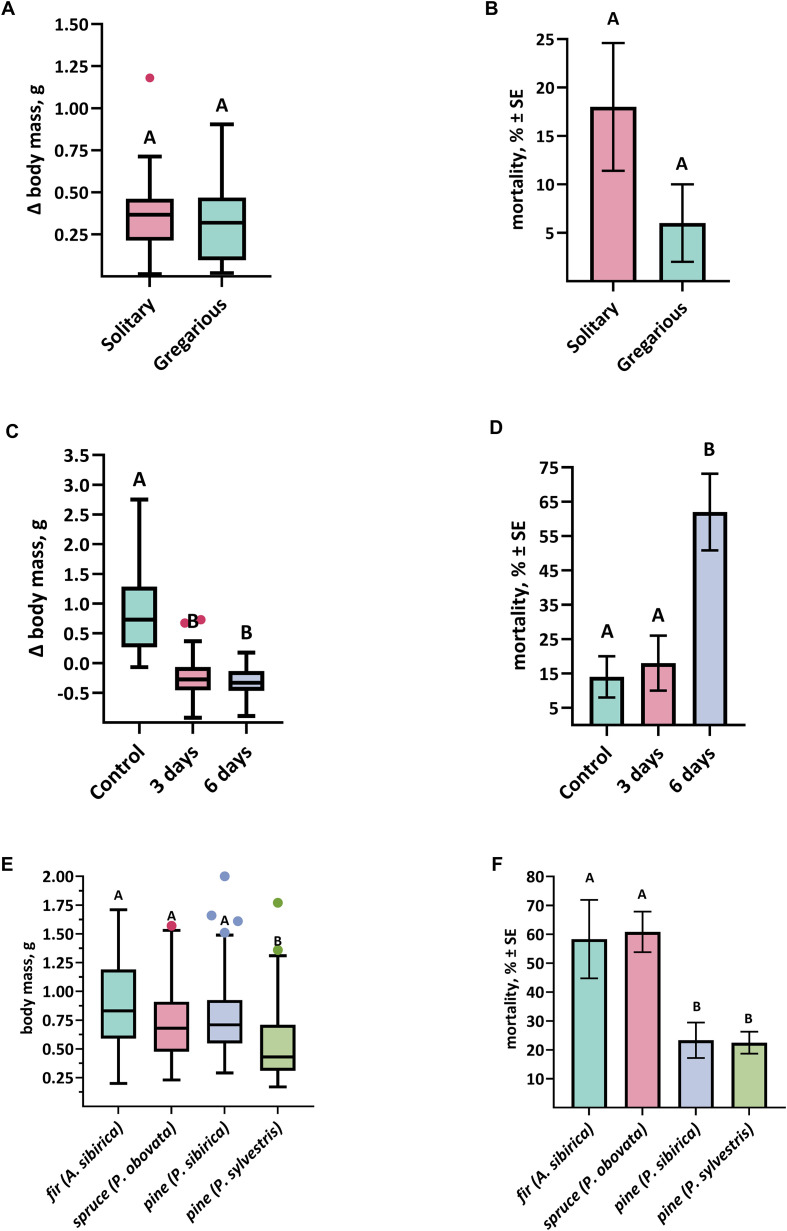
Comparison of larval body mass and larval mortality of *D. sibiricus* under **(A, B)** solitary versus gregarious conditions, **(C, D)** food deprivation for 3 and 6 days, and **(E, F)** rearing on different host plant species. Dots denote outliers. Different letters indicate significant differences (p < 0.05). See also **Figure 2** for the experimental design and group sizes.

To test whether environmental factors contribute to switching of the life-cycle program, we examined the effects of population density, food deprivation, and different host plant species (**Figures 8A–F**). In the insect population density experiment (**Figures 8A, B**), no statistically significant difference in body mass gain was observed between solitary (Δ0.30 ± 0.03 g) and gregarious (Δ0.36 ± 0.03 g) larvae (Mann–Whitney U = 822.5, p = 0.24; **Figure 8A**) ruling out high larval density as a trigger for prolonged development. No statistical differences in larval mortality was also observed (χ^2^ = 3.54, df = 1, p = 0.06, **Figure 8B**). In the food deprivation experiment (**Figures 8C, D**), a significant main effect on larval mass was found (χ^2^ = 45.3, df = 2, p < 0.001; **Figure 8C**), driven primarily by increased mass in control larvae with continuous access to food. No significant mass differences were detected between larvae deprived for 3 days vs. 6 days (Dunn’s test, p > 0.05; **Figure 8C**). However, food-deprived larvae showed signs of stress and weight loss, and larvae subjected to 6 days of deprivation exhibited significantly higher insect mortality (χ^2^ = 29.0, df = 2, p < 0.0001; **Figure 8D**). Larval mortality also differed significantly among larvae reared on different host plant species (χ^2^ = 68.0, df = 3, p < 0.0001; **Figure 8F**). The high survival rate observed on Scots pine may have been influenced by the fact that larvae were initially reared on Siberian pine until the second instar. Nonetheless, larvae fed on Scots pine had the lowest weight gain compared to those on other host plants (Dunn’s test, p < 0.001; **Figure 8E**). Despite a significant overall effect of host plant species on larval mass (χ^2^ = 41.67, df = 3, p < 0.001), all surviving larvae in the host plant experiment successfully pupated, indicating that *D. sibiricus* larvae exhibit a high degree of adaptability to diverse conifer hosts without transition to a 2-year life cycle.

## 4 Discussion

In this study, we present the first comprehensive transcriptomic investigation of ontogenetic variability in *D. sibiricus* larvae with different life cycles. Our findings reveal that the shift from a 1-year to a 2-year life cycle is accompanied by pronounced transcriptomic reprogramming with distinct tissue- and sex-specific patterns. In the head, fat body, and gut tissue sets, the 2-year life cycle larvae exhibited broad changes in gene expression relative to their 1-year counterparts. Sex-specific differences were evident only in the gut set, likely because this tissue set included also gonads and Malpighian tubules. We therefore interpret the sex-specific pattern in gut tissue set cautiously, as it may reflect reproductive transcriptional differences rather than gut-specific regulation alone. This likely explains why the sex and life cycle interaction was much more pronounced in the gut set than in the head or fat body datasets. Genes related to growth and development tended to be down-regulated in the 2-year life cycle larvae, whereas genes associated with stress tolerance, catabolism, and homeostatic maintenance were up-regulated. These results suggest that the 2-year life-cycle pathway activates an alternative program of gene expression that differs from the 1-year pathway. This pattern is associated with a diapause-like state during prolonged larval development.

GO enrichment analysis provides further insight into the physiological shifts associated with the 2-year life cycle. The DEGs identified in this study were significantly enriched in tissue-dependent patterns, with the largest number of DEGs in the male gut set (**Figure 4B**), followed by the fat body set (**Figure 4A**). The direction of change also differed by tissue sets: the male gut was dominated by broad repression of anabolic and digestive programs, whereas the female gut retained an anabolic signature; by contrast, the fat body displayed a mixed profile, with up-regulation of oxidative phosphorylation and contractile modules alongside down-regulation of ribosome biogenesis and RNA processing (**Figures 6A–C**). Together, these patterns are consistent with a partitioned seasonal strategy in which nutrient-processing tissues enter an energy-conserving state, while storage and neuromuscular functions are selectively maintained or remodeled during the shift to the 2-year life cycle. Such tissue-partitioned transcriptional differences are not unique to *D. sibiricus*. For example, in honey bees, *Apis mellifera*, a reversible diapause-like state is supported by tissue-specific programs: the fat body activates nutrient storage and stress-resilience modules, whereas wing muscle transcripts are modulated to sustain colony thermogenesis (35). One notable result was the differential expression of several cytochrome P450 domain-containing genes in the head and fat body sets of 2-year life cycle larvae (**Figure 7C**). Although P450 enzymes are often associated with xenobiotic detoxification (36, 37), cytochrome P450 proteins constitute a functionally diverse family (38). In our dataset, reduced expression of P450-related genes in the head and fat body sets is better interpreted as part of broader physiological and metabolic remodeling associated with prolonged larval development than as a direct indication of pesticide metabolism. In addition, a small set of genes annotated with ecdysteroid kinase-like domains was up-regulated in 2-year life cycle larvae (**Figure 7D**), which may be consistent with altered ecdysteroid turnover during prolonged larval development, although the underlying endocrine mechanisms remain to be clarified.

The inference of a diapause-like state is supported not only by the observed shift from growth-associated to stress-associated transcription, but also by the fact that the principal regulators highlighted in our dataset correspond to endocrine and stress-response modules repeatedly implicated in diapause across insects (25, 39–41). Similar regulatory themes have also been reported for *Drosophila melanogaster*, where dormancy-related states involve insulin/FOXO-associated metabolic remodeling, although *Drosophila* dormancy is not fully equivalent to classical diapause (22). Altered JH/Met-related signatures in 2-year life cycle larvae align with the established role of Met as a key mediator of diapause-associated JH signaling, including conserved Met-dependent regulation demonstrated in *Galeruca daurica* (43) and broader JH receptor functions reviewed by Palli and Litwack (39). Likewise, the FOXO-associated signals identified here are consistent with evidence that insulin/FOXO signaling is a central component of diapause regulation in diverse insects, including adult diapause in *Drosophila melanogaster* and *Culex pipiens* (25), as well as functional evidence from *Helicoverpa armigera*, where FOXO acts as a master regulator of pupal diapause (40). In 2-year life cycle larvae, the shift from growth to survival, marked by reduced expression of growth-promoting transcripts and increased expression of stress-response transcripts, is therefore consistent with a diapause-like state in which Met-mediated JH signaling acts in concert with FOXO (**Figures 7A, B**). The stress- and proteostasis-related component in 2-year life cycle larvae is also in line with diapause transcriptomic studies reporting enhanced cellular protection, proteasome function, and metabolic remodeling during developmental arrest (41, 42). In our dataset, Met-associated downstream modules (PI3K/AKT, STAT3, RAP1/RAC1, PTK2, and PTPN11) were prominently represented among DEGs (**Figure 7A**, except in the fat body set), and JH-binding protein domains were differentially expressed in the head set (**Figure 7E**), together indicating altered JH signaling in 2-year life cycle larvae. This interpretation is necessarily comparative, however, because it is based on similarities to diapause-associated molecular patterns described in other insects (22) rather than on direct physiological verification of classical diapause in *D. sibiricus*.

Many insect species that are capable of multiple generations per year will switch to a univoltine or semivoltine life cycle in response to cues such as photoperiod shortening or temperature decline, thereby undergoing an overwintering diapause (16, 17). *Dendrolimus sibiricus* is known to exhibit 1-year cycles in warmer or more southern locales and 2-year cycles in harsher environments (1), supporting the idea that prolonged larval development is an ecologically regulated strategy. Okunev (44) also reported that in exceptionally warm years (when cumulative degree-days above 10 °C exceed ~2,200) larvae can complete development and pupate within the same summer without overwintering (i.e. a single-season life cycle), suggesting a primary role for temperature. However, such accelerated development appears to be exceptional across most of the species’ range. In our study, larvae expressing a 2-year life cycle were occasionally observed during routine laboratory rearing at constant 23 °C temperature, but this occurred unexpectedly and outside a controlled induction experiment. By contrast, experimental manipulation of insect population density, food deprivation, and host plant species (**Figures 8A–F**) did not induce any larvae to follow the 2-year life cycle. This contrast suggests that the phenomenon is real, but that its expression probably depends on additional cues or interactions among cues that were not captured in the present experiment. Taken together, these observations indicate that initiation of the 2-year life cycle is unlikely to be explained by any single environmental factor and probably depends on a specific combination or sequence of cues and/or carry-over effects from the previous generation (e.g., parental condition or cumulative thermal exposure). Notably, Numata and Shintani (17) discussed how insect life cycles often serve as a risk-spreading strategy, allowing insects to hedge against unfavorable years or to synchronize emergence with periodically abundant resources. In *D. sibiricus*, the ability to either pupate after one summer or delay pupation until the next spring may maximize survival and eventual reproductive success under variable environmental conditions.

The transcriptomic changes observed in 2-year vs. 1-year life cycle *D. sibiricus* larvae suggest a coordinated, diapause-like reprogramming. Our results suggest that expression of the 2-year life cycle of *D. sibiricus* is not predetermined. Under increasingly favorable environmental conditions, a greater proportion of *D. sibiricus* larvae may complete development (from egg to adult) through the 1-year life cycle. Such a shift would shorten generation time at the population level and could promote more rapid increases in population density, thereby increasing the risk of repeated annual episodes of severe tree defoliation during outbreaks. In the context of ongoing climate warming, this scenario deserves particular attention, because warmer conditions may reduce the frequency of prolonged larval development and shift a larger fraction of the population toward the 1-year trajectory. By characterizing the transcriptomic profile of the 2-year life cycle in *D. sibiricus* larvae, we have preliminarily delineated candidate genetic markers (e.g., Met-mediated JH signaling components, FOXO-centered targets, and tissue-specific P450 modules) that distinguish 2-year from 1-year developmental trajectories, enabling potential molecular identification of a larva’s life-cycle state. We note that our transcriptomic analysis was limited to larval stages; future studies should examine gene expression in the pupal stage to determine whether and how the diapause-like developmental program is maintained or terminated during pupation. Unraveling these transcriptomic controls is important for understanding prolonged larval development and developmental plasticity in *D. sibiricus*. These findings clarify the transcriptomic changes associated with prolonged larval development in *D. sibiricus* and provide a basis for future studies of its physiological regulation.

## Data Availability

The original contributions presented in the study are publicly available. This data can be found here: NCBI Sequence Read Archive (SRA), accession PRJNA1451813.
